# Spatially controlled nano-structuring of silicon with femtosecond vortex pulses

**DOI:** 10.1038/s41598-020-69390-4

**Published:** 2020-07-28

**Authors:** M. G. Rahimian, A. Jain, H. Larocque, P. B. Corkum, E. Karimi, V. R. Bhardwaj

**Affiliations:** Department of Physics, University of Ottawa, K1N 6N5 Ottawa, ON Canada

**Keywords:** Optics and photonics, Laser material processing

## Abstract

Engineering material properties is key for development of smart materials and next generation nanodevices. This requires nanoscale spatial precision and control to fabricate structures/defects. Lithographic techniques are widely used for nanostructuring in which a geometric pattern on a mask is transferred to a resist by photons or charged particles and subsequently engraved on the substrate. However, direct mask-less fabrication has only been possible with electron and ion beams. That is because light has an inherent disadvantage; the diffraction limit makes it difficult to interact with matter on dimensions smaller than the wavelength of light. Here we demonstrate spatially controlled formation of nanocones on a silicon surface with a positional precision of 50 nm using femtosecond laser ablation comprising a superposition of optical vector vortex and Gaussian beams. Such control and precision opens new opportunities for nano-printing of materials using techniques such as laser-induced forward transfer and in general broadens the scope of laser processing of materials.

## Introduction

The fundamental limit to spatial resolution of any optical system is governed by diffraction and is approximately half the wavelength of light^1^. Diffraction also dictates how tightly a laser beam can be focused, which in turn determines the feature size one can achieve in laser ablation of materials. Therefore, shorter wavelengths (ultraviolet) are often used in combination with lithographic techniques to produce sub-wavelength features as small as 50 nm^2^. Driven primarily by the semiconductor industry, research efforts are ongoing to use coherent and non-coherent extreme ultraviolet light to produce features smaller than 10 nm to meet the ever increasing demand for miniaturization^3,4^. Concurrently, alternate methods are also being explored to overcome the diffraction limit of light that does not involve the use of a photomask. These fall into two categories—near field and far field approaches.

Nanofabrication using near field approach exploits local field enhancement around a nanoparticle to confine light to sub-wavelength dimensions and thereby induce local deformations (melting or ablation) of the substrate. Large scale periodic array of nanoholes were fabricated by laser irradiation of a monolayer of microspheres^5^—a multistep process with no direct control on the position of nanostructures, analogous to lithographic techniques^6^. Alternately, controlled fabrication of individual nanostructures can be achieved using scanning probe microscope either directly^7^ or by irradiating the tip with light^8^.

Direct laser processing of materials is a far field approach that exploits the nonlinear nature of the light-matter interaction and localized energy deposition. Using ultrashort laser pulses, three-dimensional (3D) control was achieved in transparent materials^9,10^ and sub-wavelength structures were created with enhanced spatial precision in a cold ablation process due to negligible lateral heat transport to the surrounding material. Exploiting near threshold ablation, feature dimensions far below the diffraction limit were demonstrated^11,12^. However, in such a threshold based material response, the ablation features were found not to be dependent on the nonlinear process responsible for light absorption but rather correspond to a one-to-one mapping of the beam profile at threshold intensity^13^. Nanoholes^14,15^, nanocones^16–19^, nanodots^5^ and self-organized periodic nano-ripple patterns^20–22^ were also fabricated in different materials either by ablation or material modification using above threshold laser pulse energies.

Another non-contact direct laser-write technique that is widely used in nano-printing is Laser Induced Froward Transfer (LIFT) to “drop and place” small volumes of complex materials into user-defined, high-resolution patterns^23,24^. A thin film of material on a donor substrate is melted locally by the laser beam and lifted off in the form of a droplet that gets deposited onto a receiver substrate separated by a small gap. The spatial resolution one could achieve in placing individual droplets is few hundreds of nanometers.

Although light induced nanostructures with feature sizes smaller than diffraction limit could be fabricated either by ablation or material modification^25^, the degree of spatial control with light is restrictive. For example, it is not feasible to position individual structures with nanoscale precision. Only partial spatial control has been achieved with nano-ripples whose orientation and spacing was varied by changing the laser polarization and wavelength ($\lambda$)^21^. In this context, the present article addresses how light can be used to actively manipulate materials in two dimensions with a precision of $\sim \lambda$/20. It demonstrates positioning of $\sim$ 0.1 $\upmu \hbox {m}^3$ of the molten material in the form of a nanocone with 50 nm precision in an area of 40 $\upmu \hbox {m}^2$ by manipulating the beam shape. The technique is extended to fabricate complex, unconventional structures involving multiple nanocones and control their relative positions with the same precision.

Apart from laser fabrication and spatial control of nanostructures, surpassing the diffraction barrier imposed by the wave nature of light is also critical in imaging/microscopy. It is accomplished by using the same (a) near field techniques that exploit the information contained in the evanescent wave^26,27^ or confine light using plasmonic nanostructures^28,29^, and (b) far field techniques that exploits the optical nonlinearity of the medium as in stimulated emission depletion microscopy^30^.

Our approach to sub-wavelength precision in nano-fabrication is based on the coherent superposition of optical vector vortex beam (VVB) and a Gaussian beam^31^. VVBs are characterized by spatially variant linear polarization in the beam transverse plane. They may possess phase singularities in the transverse plane at which the field amplitude vanishes. VVBs can be expanded in terms of orbital angular momentum (OAM) carrying, i.e. twisted, beams. Twisted beam carry an OAM value of $\ell \hbar$ per photon, where $\hbar$ is the redueced Plank constant, and $\ell$ indicates the number of twists in the helical wavefront in one wavelength which its sign determines the chirality of the helix. We produce VVBs using a birefringent plate enclosing a patterned liquid crystal layer, known as a ${q}$-plate. The liquid crystals in the ${q}$-plate have an optic axis whose orientation depends on the azimuthal coordinate, thereby forming a pattern defined by a topological charge *q* consisting of either a full or a half-integer value. As light propagates in the ${q}$-plate, spin angular momentum associated with light polarization is coupled to photon OAM of $\ell = \pm 2 {q}$. The conversion efficiency is determined by the ${q}$-plate’s optical retardation, which can be controlled by an externally applied electric field.

## Results

**Figure 1:**
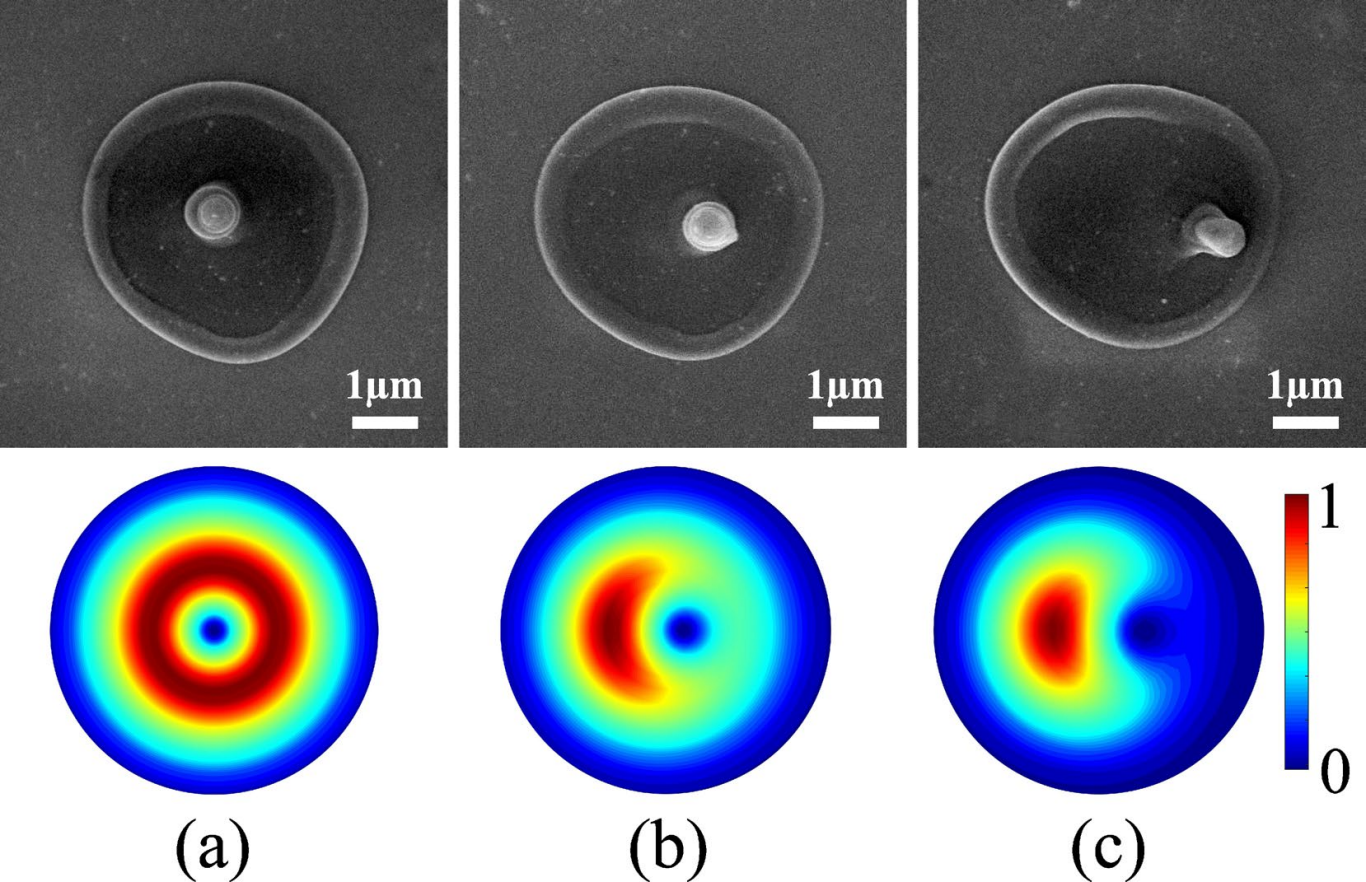
Displacement of the nanocone and phase singularity with retardation. Superposition of linearly polarized Gaussian beam with VV beam produced by a *q*-plate with topological charge of $q=+1/2$, having different weights (**a**) 0:100 (pure VV beam), (**b**) 10:90, and (**c**) 15:85. The top row shows SEM images of the nanocone position and the bottom row shows the corresponding intensity profile of the superposition beams. A single laser pulse with an energy of 280 nJ created the nanocone. The corresponding peak fluence is 2.3 ± 0.3 J/$\hbox {cm}^2$.

Our technique exploits two unique properties of VVBs. First, the annular intensity distribution of the VVB when focused is mapped onto the silicon surface causing melting of a thin layer, determined by the optical penetration depth of light. Thermo-capillary and/or hydrodynamic forces displace the molten silicon radially outward to the periphery and also radially inward to the centre of the crater. Compressive forces arising from radial inward motion of the molten material pushes it away from the surface. Rapid expansion causes re-solidification into a nanocone formed (Fig. 1a) at the centre where VVB has a zero intensity point^18^. Simultaneously, the radial outward motion of the molten material re-solidifies after reaching the cold boundary of the ablation region to form a rim. The height of the nanocone is $\sim$ 500 nm and increases with the increasing pulse energy while the rim height is only $\sim$ 100 nm^18^. Longer nanocones or nanoneedles have been produced with vortex beams using nanosecond and picosecond pulses on silicon^32^ and metals^33^. They were shown to exhibit chirality and has been attributed to (a) mapping of the orbital angular momemtum of the beam on to the handedness of the nanoneedles^33^, and/or (b) tailored chiral intensity distribution that also controls the handedness of nanoneedles^34^. In contrast, there was no clear signature of the chirality-control fabrication (by changing OAM value) of nanocones produced by femtosecond pulses.

Second, when a pure VVB is perturbed by adding coherently a tunable amount of a linearly polarized Gaussian beam, the central singularity either shifts or unfolds into multiple singularities depending on the topological charge. The shift can be precisely controlled by adjusting the applied external field to the ${q}$-plate. This action varies the optical retardation and thereby detunes the strength of the spin-to-orbital angular momentum coupling of light. A fraction of the input Gaussian beam co-propagates with a partly converted VVB. As a result, for ${q}$=1/2 or $\ell = \pm 1$, the position of the nanocone within the ablation region can be varied with nanometers precision (Fig. 1b,c). As the weight of the Gaussian beam increases, the position of the singularity shifts towards the outer region of the ablated region (also see Supplementary Fig. S2). For a pure VVB produced by ${q}$=1/2 plate, the output polarization was radial, azimuthal, or spiral^22^ when the angle of the incident linear polarization with respect to the *q*-plate axis was 0$^\circ$, 90$^\circ$, or 45$^\circ$, respectively.

**Figure 2:**
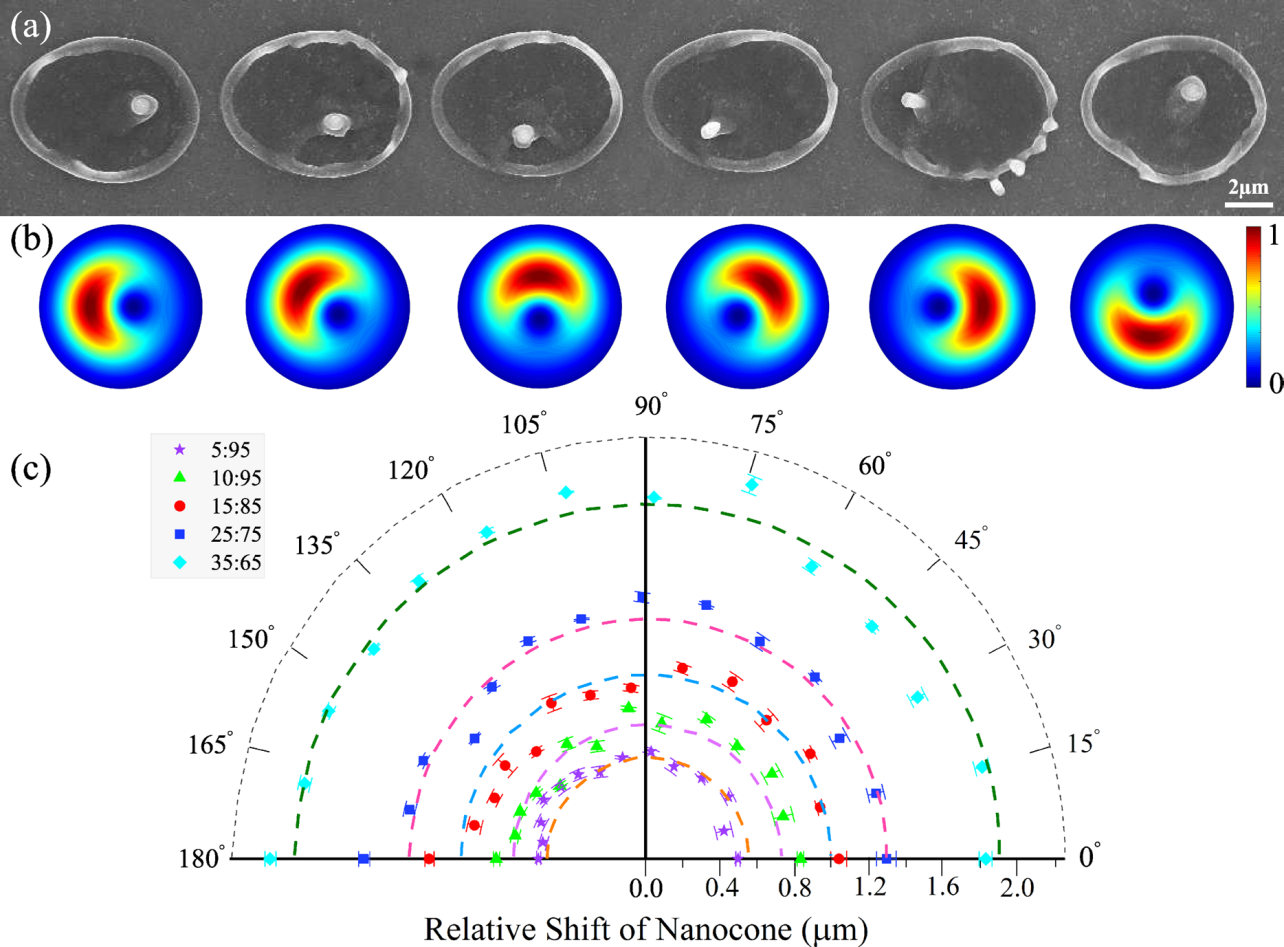
Controlled positioning of the nanocone in 2D space. (**a**) For a fixed relative weight (25:75) of Gaussian and VV beams produced by an electrically detuned ${q}$-plate with topological charge of ${q}$=$+1/2$, SEM images show the motion of the nanocone in a circular arc when an additional phase is added to the superimposed beams by rotating the polarization axis of the incident Gaussian beam with respect to the ${q}$-plate axis. A single laser pulse irradiated the sample with a pulse energy of 600 nJ. (**b**) The corresponding simulated intensity profiles. (**c**) Polar plot showing the measured relative shift of the nanocone with respect to its position for a pure VV beam as a function of the rotation angle of the HWP (relative phase of the linearly polarized Gaussian beam) for different optical retardations (different weights of Gaussian and VV beams). The dashed lines are the simulated nanocone position.

The nanocone position can be controlled by moving the singularity anywhere in the transverse plane of the beam as shown in Fig. 2, for azimuthally polarized VVB. For a fixed optical retardation (relative weight of Gaussian beam to VVB), a half-wave plate (HWP) in front of the detuned ${q}$-plate rotates the input polarization with respect to the ${q}$-plate axis and changes the angular position of the singularity at the transverse plane rotating it in a circular arc. Radius of the circular arc depends on the optical retardation. Varying the applied electric field to the ${q}$-plate and rotating the incident linear polarization, the nanocone can be positioned anywhere in 2D space within the interaction region of $\sim$ 40 $\upmu$ $\hbox {m}^2$ with a precision of 50 nm. Similar results were obtained for radial and spiral VVBs.

**Figure 3:**
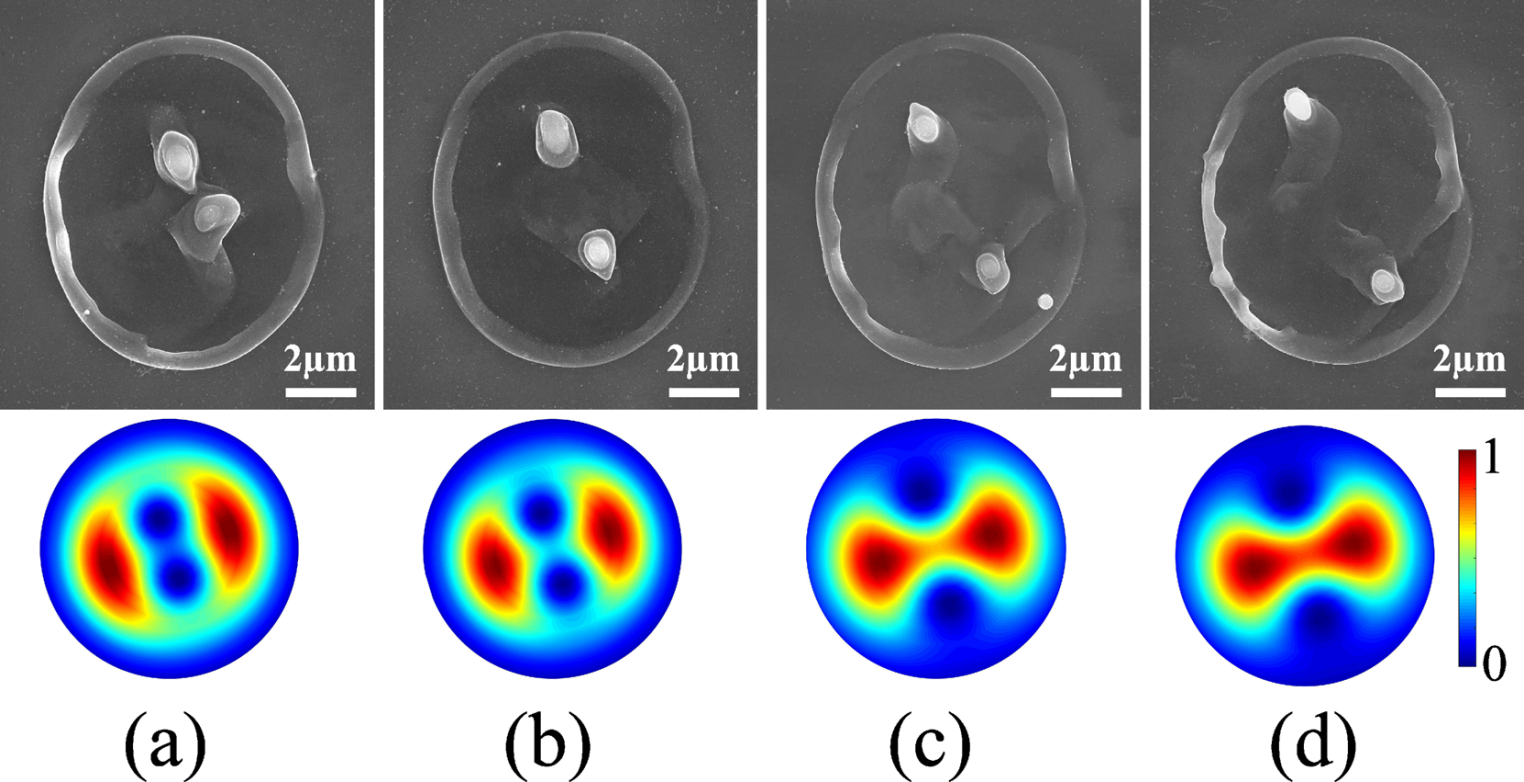
Separation of nanocones with unfolding singularity. Superposition of linearly polarized Gaussian and VV beams produced by a detuned *q*-plate, topological charge of $q=-1$, with different relative weights of (**a**) 10:90, (**b**) 15:85, (**c**) 20:80 (**d**) 25:75. Top panels shows the SEM images of two nano-cones generated by a single laser pulse with an energy of 310 nJ. The bottom panels show the corresponding intensity profiles. Separation between the singularities increases with increase in the Gaussian component.

Figure 3 shows unfolding of the singularity when a pure VVB with a star-shaped polarization pattern^22^ with $\ell = \pm 2$ (topological charge of ${q}$ = $-1$) is perturbed by a Gaussian beam. The perturbation is achieved by electrically detuning the ${q}$-plate. In VVBs, the total field orientation is undefined along the beam axis and this polarization singularity is typically referred to as a V-point. A small perturbation to pure VVB causes the local polarization states to acquire a tiny ellipticity. The V-point no longer exists, instead two pairs of C-points appear where the orientation of the polarization ellipse is undefined. Unfolding of the polarization singularity also deforms the intensity pattern of the pure VVB giving rise to two null intensity points. The separation between these points can be controlled by varying the relative weight of Gaussian beam superimposed on the VVB and increases with the amount of Gaussian contribution. This results in the formation of two nanocones whose separation and relative orientation can be precisely controlled by detuning the *q*-plate and adding an extra phase to the superimposed beams (as in Fig. 2), respectively (see Supplementary Fig. S4).

**Figure 4:**
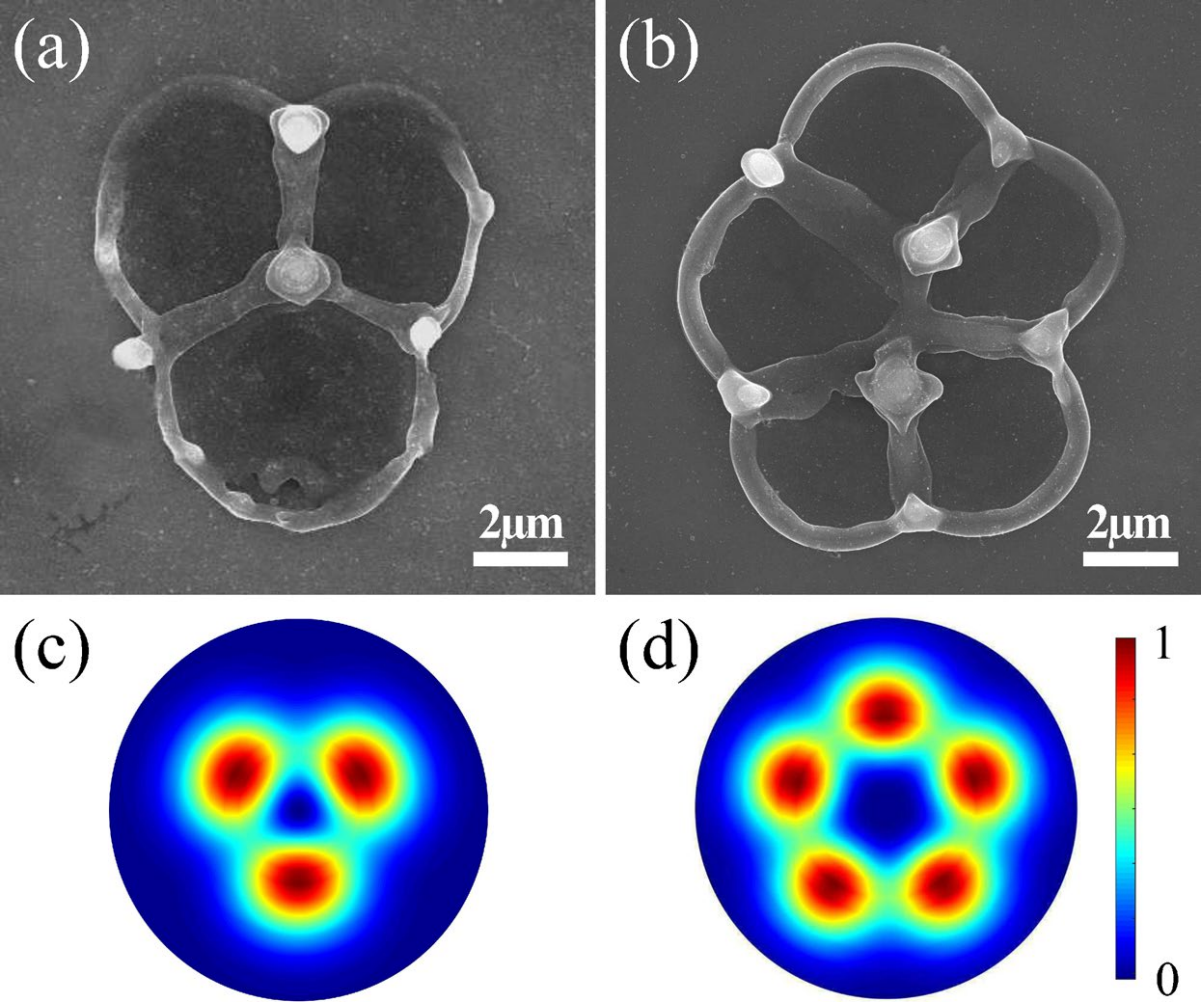
Fabrication of complex nanostructures. Mapping of complex intensity profiles generated by VVB produced by two *q*-plates with topological charge of $q=-1$, and $q=1/2$. (**a**) A HWP between the $q=-1$ ($\ell = \pm 2$) plate and a detuned $q=1/2$ ($\ell = \pm 1$) plate, and (**b**) without the HWP between the two *q*-plates. The corresponding calculated intensity profiles are shown in (**c**,** d**). Experimental patterns were produced by a single pulse with an energy of 700 nJ.

Figure 4 shows how complex intensity patterns can be generated by superimposing different VVBs produced by a combination of two *q*-plates with topological charges of $q=-1$ and $q=1/2$. A HWP between the $q=-1$ and a detuned $q=1/2$ plates produces a superposition state $\alpha$($\hbox {e}^{i2\phi }$ ${\mathbf {e}}_R$ + $\hbox {e}^{-i2\phi }$ ${\mathbf {e}}_L$) $+$ $\beta$($\hbox {e}^{i\phi }$ ${\mathbf {e}}_L$ + $\hbox {e}^{-i\phi }$ ${\mathbf {e}}_R$), where $\alpha$ and $\beta$ are given by the detuning parameter. The resultant intensity distribution displays three singularities around the central region where the intensity is also minimum (Fig. 4c). As a result 4 nanocones are formed (Fig. 4a). In the absence of the HWP the output corresponds to a superposition state defined by $\alpha$($\hbox {e}^{i2\phi }$ ${\mathbf {e}}_R$ + $\hbox {e}^{-i2\phi }$ ${\mathbf {e}}_L$) $+$ $\beta$($\hbox {e}^{i3\phi }$ ${\mathbf {e}}_L$ + $\hbox {e}^{-i3\phi }$ ${\mathbf {e}}_R$). The intensity pattern consists of 5 null points around the central null region (Fig. 4d). This should lead to 6 nanocones. However, any slight detuning of the $q=-1$ plate leads to splitting of the central singularity resulting in the formation of 7 nanocones (Fig. 4d) (see Supplementary Fig. S6).

## Discussion

There are some similarities between our laser processing technique and stimulated emission depletion (STED) microscopy/ lithography in surpassing the diffraction barrier. Both use superpositions of Gaussian and non-Gaussian beams. In STED microscopy, a focused Gaussian beam that excites the fluorophores is superimposed with a beam having a doughnut intensity distribution that switches off the fluorophores except at its centre. In STED lithography the doughnut beam is used to inhibit photo-polymerization induced by the writing beam. However, the main drawback of STED lithography is the design and development of suitable photoresists^35^. In our method, varying the phase retardation between the VVB and Gaussian beam shifts the null intensity positions within the focal region enabling us to control the position of the nanocone.

However, the underlying physics is different between the two methods. In STED microscopy, the doughnut beam depletes the fluorescent state by stimulated emission in all molecules in the intense regions of the beam. The intensity of the doughnut beam determines (a) the probability of fluorescence switching that scales exponentially, and (b) the area to which fluorescence is confined that scales inversely thereby enhancing the spatial resolution. In our technique, VVB induces localized melting via multiphoton absorption and the subsequent fluid dynamics around the unmodified region of the material (due to null intensity region) displaces matter on nanoscale. The probability of such nonlinear interaction scales with $n^{th}$ power of laser intensity, where *n* is the number of photons involved in the multiphoton process. In silicon (band gap of 1.14 eV), the interaction of 800 nm light (photon energy of 1.55 eV) is dominated by single and two photon absorption. The intensity of the VVB determines the amount of the material displaced leading to nanocone formation whose height increases but the apex angle remains the same^18^. Therefore, the spatial resolution remains the same.

In our experiments, the spatial precision of $\sim$ 50 nm is due to loose focusing of the laser beam. The use of a high numerical aperture lens will reduce the size of the null intensity region and will lead to a smaller lateral extent and apex angle of the nanocone (see Supplementary Fig. S3). However, at very high numerical apertures the longitudinal component of the field can hinder the interference process and limit the spatial resolution. The spatial precision can be further improved to 10 nm by (a) using a highly stable power supply that can change the small voltage applied to the *q*-plate in 1 mV steps, and/or (b) changing the angle of the half-wave plate in front of the detuned *q*-plate in smaller steps of 0.1$^\circ$. The laser pulse duration is not critical in our technique. In fact, longer durations were found to give rise to $\upmu$m sized nano-needles likely due to larger melt volume^19^.

The main limitation is that the nanocones can be efficiently created only on non-transparent materials where the melt layer is two dimensional. In transparent materials, light penetration into the medium leads to a 3D melt layer whose dynamics lead to surface swelling and ejection of the material instead of a well-defined nanocone. Also, the nanocones are always accompanied by an outer rim whose relative height can be minimized by laser parameters albeit with concomitant reduction in nanocone height^18^. Controlled fabrication of nanocones in semiconductors and metals can be used as field emission tips^36,37^, scanning probes, whispering gallery optical resonator^38^, and for enhanced solar absorption in photovoltaics^39^. When implemented with LIFT, our technique provides the ability to control the deposited material with sub-wavelength precision in nano-printing of complex materials with applications ranging from microelectronics to bio-photonics.

## Methods

### Experiment

Femtosecond light pulses from a Ti:sapphire laser system (800 nm, 1 kHz, 45 fs, 2.5 mJ/pulse) were focused on a silicon surface with a 0.25 NA (16X) aspheric lens. The sample was mounted on three-axis translation stages with a resolution of 100 nm. The sample was irradiated with a single pulse selected by operating the laser in an external trigger mode. The incident pulse energies, varied using a half-wave plate (HWP) and a polarizer, were measured after the microscope objective taking into account the transmission and reflection losses of all the optics. The pulse duration before the microscope objective was 70 fs. The laser-ablated regions were characterized by a scanning electron microscope (SEM), with the electron beam incident normal to the sample surface and atomic force microscopy (AFM) in non-contact mode.

In our experiment, complex intensity profiles were generated using birefringent-based liquid crystal beam converters, called *q*-plates^40,41^, with topological charges of $q=+1/2$ and $-1$. The optical retardation of the *q*-plates was changed by varying the voltage applied to them. At the optimal voltage, the *q*-plates converted linearly polarized Gaussian beams to optical VVBs composed of OAM states with $\ell = \pm 1$ and $\ell = \pm 2$, respectively^42^. Complex spatial intensity profiles were produced by varying the voltage applied on the *q*-plate. This process called voltage tuning of the *q*-plate results in varying the extent of coherent superposition of laser beam components. In other words, detuning the individual *q*-plate produced a superposition of partially converted VVBs with the incident Gaussian beam. A combination of different *q*-plates resulted in complex intensity profiles due to the superposition of VVBs. See Supplementary Fig. S1 for additional details.

### Numerical

Intensity profiles of different order VVBs and their superposition states were simulated using the Laguerre Gaussian beam

1 $$\begin{aligned} \begin{aligned} U(r,\phi ,z) =\frac{C_{\ell p}^{LG}}{w(z)}\Bigg (\frac{r\sqrt{2}}{w(z)}\Bigg )^{\mid \ell \mid }exp\Bigg (\frac{-r^2}{w^2(z)}\Bigg )L_p^{\mid \ell \mid }\Bigg (\frac{2r^2}{w^2(z)}\Bigg ) exp\Bigg (\frac{-ikr^2}{2R(z)}-i\ell \phi -ikz+i\Psi (z)\Bigg ), \end{aligned} \end{aligned}$$

where $\displaystyle p\ge 0$ is the radial index and $\ell$ is the azimuthal index. $\displaystyle L_{p}^{\ell }$ are the generalized Laguerre polynomials and $\displaystyle C_{\ell p}^{LG}$ is a normalization constant. $\displaystyle R(z)$ is the radius of curvature of the wavefront, $\displaystyle w(z)$ is the beam width and $\Psi (z)$ is the Gouy phase. $\displaystyle r$ and $\displaystyle z$ are the radial and axial distances. $\displaystyle k$ is the wave number and $\displaystyle e^{i\ell \phi }$ is the phase factor containing the $\ell$ term.

## Supplementary information

Supplementary Information 1.
