# Supplementary material for: Spatially controlled nano-structuring of silicon with femtosecond vortex pulses
Source: Sci Rep. 2020 Jul 28;10:12643. doi: 10.1038/s41598-020-69390-4 (PMC7387531; doi:10.1038/s41598-020-69390-4)
Supplement: Supplementary file 1 — Supplementary Information 1. [file 41598_2020_69390_MOESM1_ESM.pdf]

Spatially controlled nano-structuring of silicon with femtosecond vortex pulses

M. G. Rahimian, A. Jain, H. Larocque, P. B. Corkum, E. Karimi, V. R. Bhardwaj
Department of Physics, University of Ottawa, 25 Templeton Street, Ottawa, K1N 6N5, Canada

I. EXPERIMENTAL SETUP

A schematic of the experimental setup used to demonstrate spatially controlled formation of nanocones on silicon with femtosecond vortex pulses is shown in Fig. S1. Linearly polarized input pulses with a Gaussian spatial profile were converted to optical vortex pulses by employing q-plates with topological charges of $q = +½$ and -1. The resultant radial profile of the vector vortex (VV) pulse is similar to that of Hyper-Geometric Gaussian modes [1].

The q-plate is a slab of a birefringent material based on liquid crystal technology with uniform birefringent phase retardation (δ) and a transverse optical axis pattern with a topological charge of q [2]. The optical phase of the generated modes varies by $2\pi q$ when circling once the beam axis. The topological charge q is an integer or semi-integer, positive or negative value. A circular aperture after the q-plate filtered the central part of the beam resulting in a vortex beam with an annular spatial profile similar to that of Laguerre-Gauss modes.

Different polarization states with a variety of spatial intensity distributions were produced by (i) using a combination of different q-plates and wave plates, and (ii) varying the optical retardation of the q-plate, resulting in the superposition of pure vortex and Gaussian beams. The pulse energies of the emerging vortex beam with complex polarization and spatial structure were controlled by a combination of half wave plate (HWP) and polarizer. A glass plate (G) reflected a portion of the incident pulse on to a fast photodiode (PD) that was used for power calibration by operating it in the linear regime with the help of neutral density filters (ND).

An aspheric lens (16x with 0.25 NA, f = 11mm, Newport) focused the vortex pulses on to a crystalline (100) p-type silicon mounted on a precision 3-axis (x, y, z) motion control system. The position of the laser focus relative to the sample's surface was determined accurately (±5 μm) by imaging the back-reflected light from the silicon surface. Glass plate directed the back-reflected light towards a charge-coupled-device (CCD) camera (MCE-B013-US, Mightex) after propagating through a focusing lens (Thorlabs, f = 100 mm, plano-convex lens). The Si surface morphology was analyzed using a field emission scanning electron microscope (Zeiss Gemini SEM 500) with both In-Lens detection mode (top-view images) and secondary electrons detection mode (side–view images) and a Park NX10 atomic force microscope in a non-contact mode.

The setup shown in Fig. S1(a) produces a nanocone whose position can be shifted by changing the voltage applied on the q-plate with a topological charge of ½ (QP1). This corresponds to Fig. 1 in the main text. A half-wave plate in front of the QP1, as shown in Fig. S1(b), enables to change the angular position of the nanocone. This corresponds to Fig. 2 in the main text. A q-plate with topological charge of -1, QP2, as shown in Fig. S1(c) is used to produce two nanocones whose separation can be varied by changing the voltage applied on the

QP2. This corresponds to Fig. 3 in the main text. The setup shown in Fig. S1(d) produces three-petal shaped flower like structure with four nanocones, corresponding to Fig. 4a in the main text. Removing the half-waveplate between QP2 and QP1, as shown in Fig. S1 (e), produces five-petal shaped flower like structure with 6 nanocones. This can be increased to 7 nanocones by detuning QP2, corresponding to Fig. 4b in the main text.

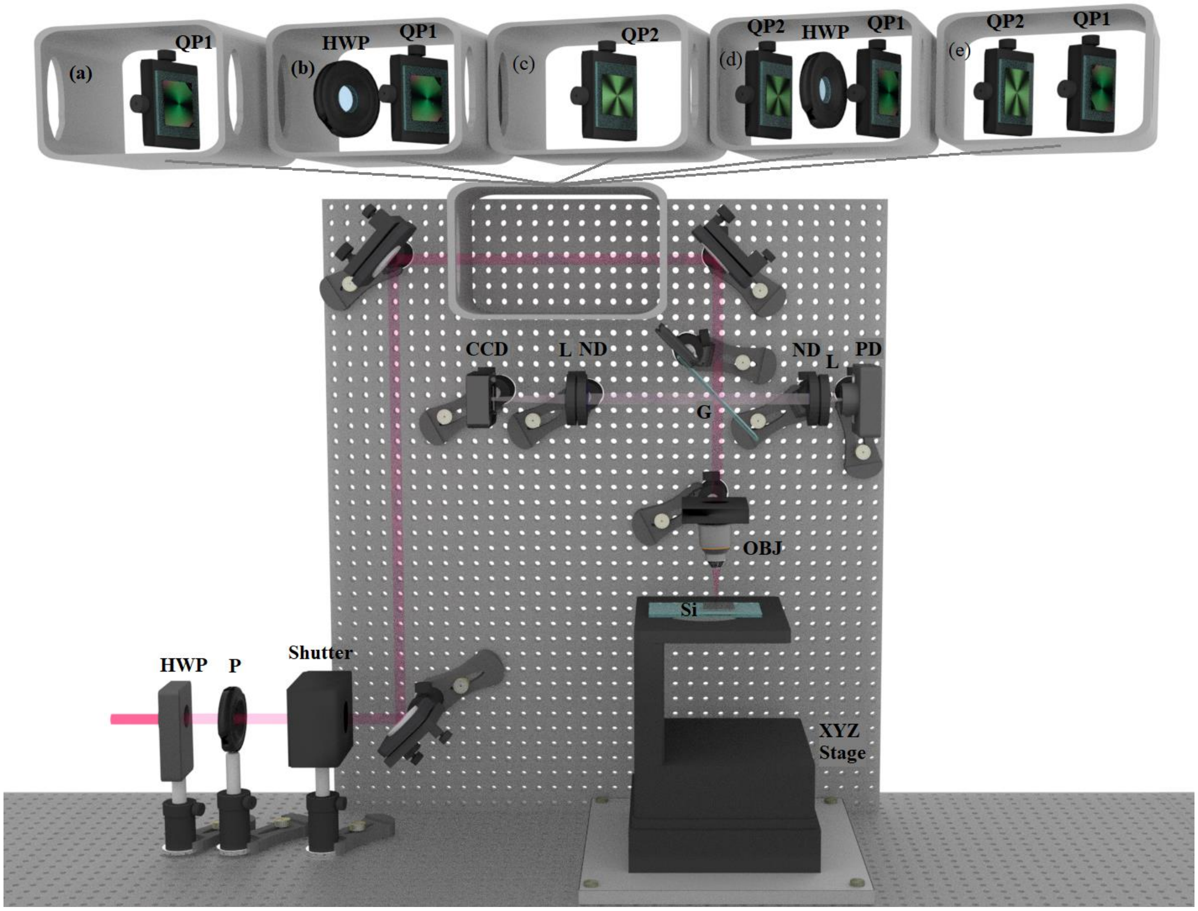

Figure S1. The schematic shows the experimental setup. ND: neutral density filter, CCD: charge-coupled-device camera, G: glass plate, PD: photodiode, P: polarizer, L: Lens, OBJ: objective, QP1: *q*-plate with topological charge of ½, QP2: *q*-plate with topological charge of -1, HWP: Half-wave plate.

II. OPTICAL RETARDATION CALIBRATION WITH THE APPLIED VOLTAGE

A square-wave signal generator operating at 7 KHz and 4 KHz was used to adjust the driving voltages to the q = +½ and q = -1 plates, respectively. At optimal voltage of 2.97V, both q-plates produced pure VV beams. Varying the voltage on the q-plates causes a change in the optical retardations (δ) enabling the tuning of the q-plates. When the q-plate is detuned, if the input Gaussain beam is circularly polarized, the resultant output beam will be a coherent superposition of two fundamental optical states; (i) the unconverted part of the input Gaussian beam with the same polarization as the input and (ii) the converted annular VV beam with opposite handedness to the input polarization with ℓ= ±1(or ±2). Invoking different input polarization states, optical

retardation values, and/or different location of polarization optics within the setup produced a combination of converted and unconverted parts of the beam.

In a simple configuration, with only q = ½ plate in the setup we were able to control the relative position of the nanocone with respect to its original location (produced by a pure vortex beam with $\ell = \pm 1$). For a fixed relative phase between the LG beam components, varying the optical retardation shifted the nanocone as shown in Fig. S2. This was achieved by detuning the voltage on the q-plate. As the relative weight of the Gaussian beam increased with respect to a pure VV beam (voltage decreased) the vortex and hence the nanocone position shifted linearly away from the center of the ablated region and moved towards the boundary (rim). The first data point corresponds to the nanocone's position when the composite beam is dominated by the Gaussian component while the last data point corresponds to a pure VV beam.

The following procedure illustrates how the optical retardation was calibrated with the voltage applied on the q-plate. Left-circular (L) polarized light was passed through the q = ½ plate to achieve spin to orbital angular momentum conversion. After the q-plate, a quarter wave plate (QWP) whose optics axis is at 45° with respect to the q-plate and a polarizing beam-splitter (PBS) produced two output beams; a pure converted beam with a doughnut-shaped profile as a transmitted component, and an unconverted beam with a Gaussian profile as a reflected component. The transmitted component corresponds to the pure VV beam with right circular polarization (R, ℓ=1) while the reflected component corresponds to pure Gaussian beam with left circular polarization (L, ℓ=0).

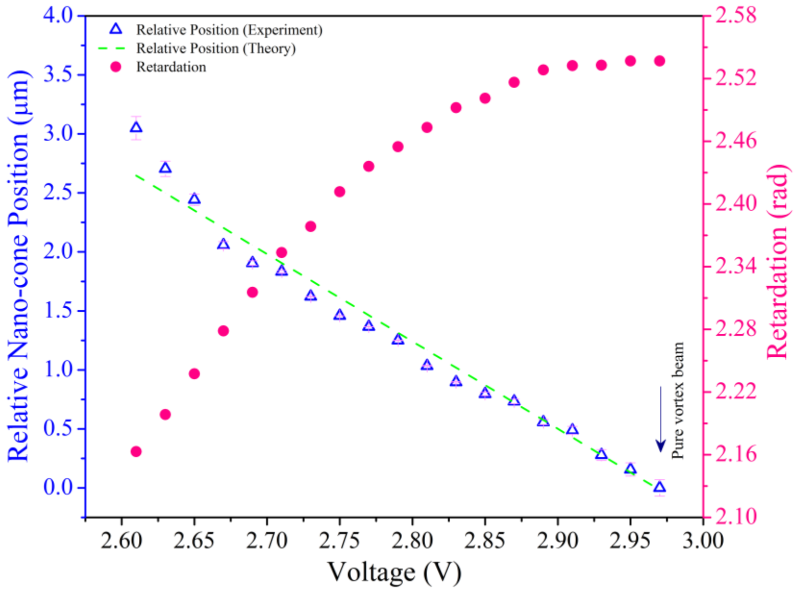

Figure S2. The left ordinate in blue shows relative position of the nanocone as a function of voltage on the q-plate, measured with respect to its original location when a pure OAM beam irradiated the silicon surface. The green dashed line corresponds to the theoretical value demonstrating linear dependence. Right ordinate in pink demonstrates variation of optical retardation with applied voltage.

For a total power of P_0, the powers of the coherently converted ($P_{R,1}$) and unconverted ($P_{L,0}$) components depend on the optical retardation as,

$$P_{R,1} = P_0 \sin^2\left(\frac{\delta}{2}\right)$$
$$P_{L,0} = P_0 \cos^2\left(\frac{\delta}{2}\right) \qquad \text{(S1)}$$

Varying the applied voltage to the *q*-plate and measuring the power of the two output beams of the PBS determined the optical retardation. The torque exerted on the liquid crystal molecules by the applied electric field is responsible for such dependency [3].

III. NA=0.25 VERSUS NA=0.55

Spatial precision of ~ 50nm was achieved in placing the nanocone within the ablated region by focusing vortex pulses with a numerical aperture (NA) of 0.25, determined primarily by the dimensions of the nanocone tip. The spatial precision can be improved further by tighter focusing with a high NA lens. This creates a smaller null intensity region leading to a smaller/sharper nanocone as shown in Fig. S3. The graph shows two nanocones with radii of curvature of 30 nm and 80 nm produced by aspheric lenses with NA=0.25 and NA=0.55, respectively. The results confirm that the radius of curvature of the nanocone tip decreases as the numerical aperture of the objective increases. Figure S3(b) presents a side-view SEM image of a nanocone produced by a single vortex pulse with an energy of 400 nJ.

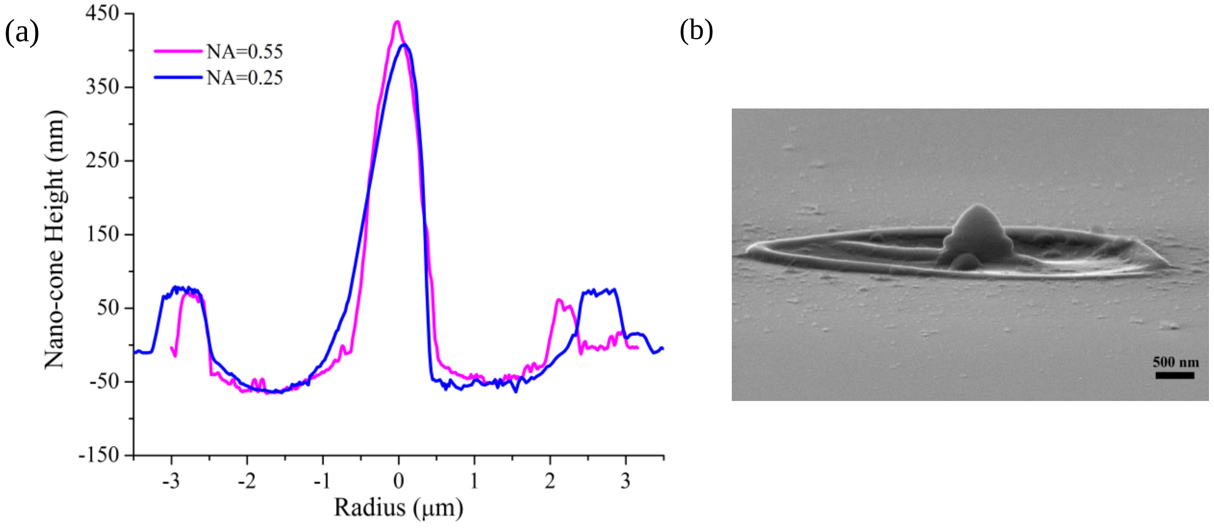

Figure S3. (a) The AFM measurements for two nanocones produced by aspheric lenses with NA=0.25 and NA=0.55. The radii of curvature of 30 nm and 80 nm were achieved, respectively. (b) A side-view SEM image of a nanocone created by a 400 nJ vortex pulse.

IV. NANOCONES SEPARATION VS OPTICAL RETARDATION

When a pure VV beam of ℓ=2 is perturbed, two nanocones are formed within the ablated region. Their separation can be precisely controlled by detuning the *q*-plate as shown in Fig. S4. In a detuned *q*-plate, Gaussian beam is superimposed on the VV beam. Such superposition results in decomposition of the vortex into two single-charged vortices (each dark spot with charge of ℓ=1). Varying the voltage on the *q*-plate changes the relative weights of the Gaussian and vortex components. In other words, the optical retardation hence the separation between the nanocones changes.

Figure S4 shows the nanocones separation decreases as the portion of the Gaussian beam decreases or the optical retardation increases. The orientation of the two nanocones can also be precisely controlled by varying the relative phase between the two components of the superimposed beams, as shown in Fig.2 of the main text. This can be achieved by rotating a HWP introduced before the *q*-plate.

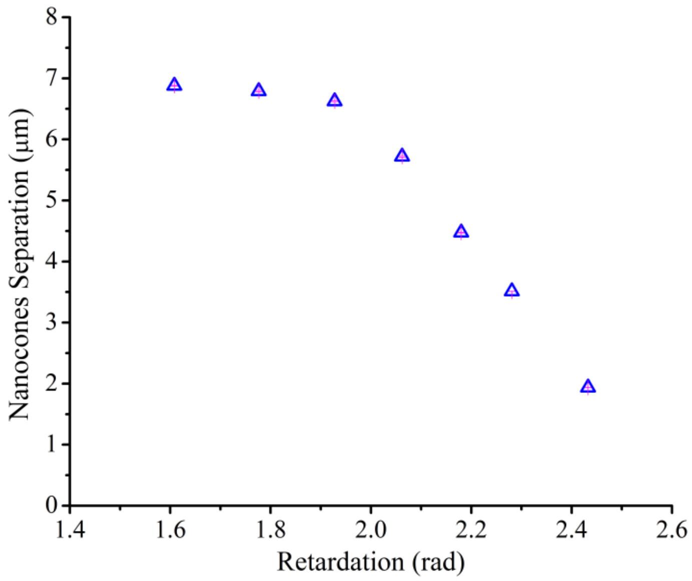

Figure S4. Decrease in nanocones separation as the optical retardation of q = -1 plate is increased. For a tuned *q*-plate, only one nanocone exists at the center of the ablated region. When *q*-plate is detuned, optical retardation decreases leading to an increase in the weight of the Gaussian beam and the nanocones separation.

V. MANIPULATING THE NUMBER AND POSITION OF THE NANOCONES

Optical retardation and topological charge of the *q*-plate play a crucial role in determining the number and the location of the nanocones. For a fixed topological charge, changing the optical retardation changes the relative weight of the optical states present in the coherent superposition leading to different asymmetric intensity distributions. For instance, a three-petal flower intensity profile was produced by an experimental configuration consisting of a *q*-plate with topological

charge of q = -1 followed by a HWP and a q-plate with topological charge of q = ½ . Varying the voltage applied on the second q = ½ plate while the applied voltage on the first q = -1 plate was fixed for a maximum conversion ($\delta = \pi$), allowed us to set the intensity ratio and achieve several position-controlled nanocones. In this case, a superposition of two beams with ℓ = +1 and ℓ=-2 was generated which allowed to have an optical vortex of charge ℓ= +1 at the centre surrounded by three vortices of charge -1 arranged symmetrically and located at the same radial distance from the centre at the angles of π/3, π, and 5π/3 [4]. As the relative amplitude of the ℓ=-2 component in the superimposed beam was decreased (increased), the peripheral vortices and hence the nanocones positions moved radially in (out), as shown in Fig. S5.

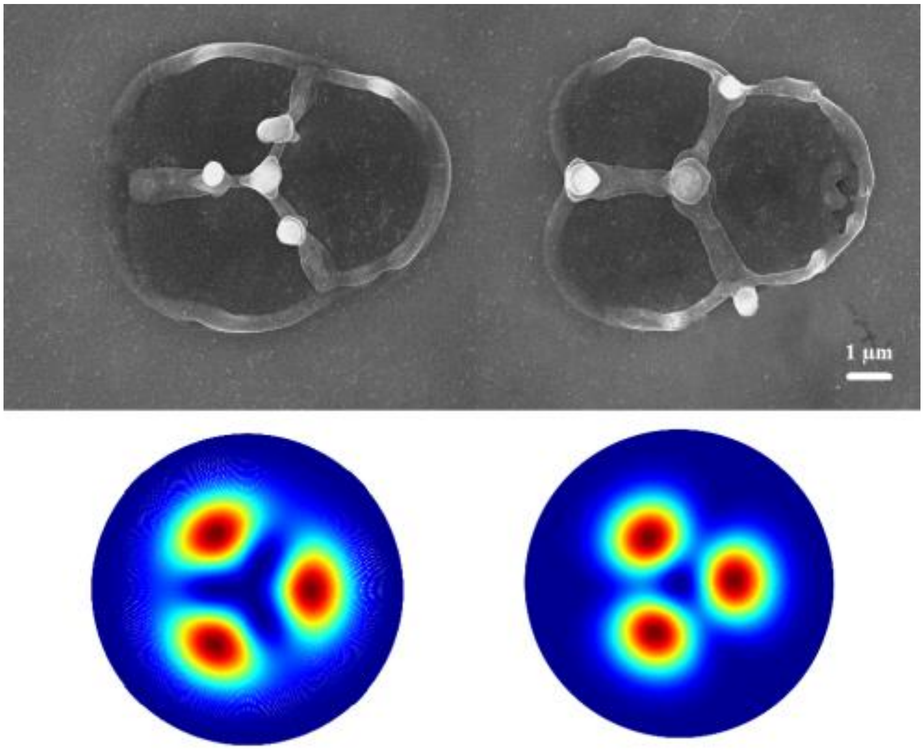

Figure S5. (Top panel) the SEM images show the peripheral nanocones moving radially outward as the relative amplitude of the ℓ=-2 component in the superimposed beam increased. Three nanocones are located at the same radial distance from the centre at the angles of π/3, π, and 5π/3. (Bottom panel) Simulation of intensity profiles corresponding to the SEM images.

We were also able to generate a five-petal flower intensity distribution by using a sequence of a HWP, a tuned q-plate with topological charge of q = -1, and a q = ½ plate with an adjustable voltage. When the alignment is accurate, the vortex laser beam regenerated in this configuration produced a structure consisting of a single nanocone at the centre and five peripheral nanocones located at the same radial distance from the central cone, as shown in Fig. S6(a). A small detuning of the first q-plate with topological charge of q = -1 resulted in the central nanocone to split into two, as shown in Fig. 4b of the main text. The corresponding AFM image is shown in Fig. S6(b). The nanocones height varies between 400 nm and 670 nm.

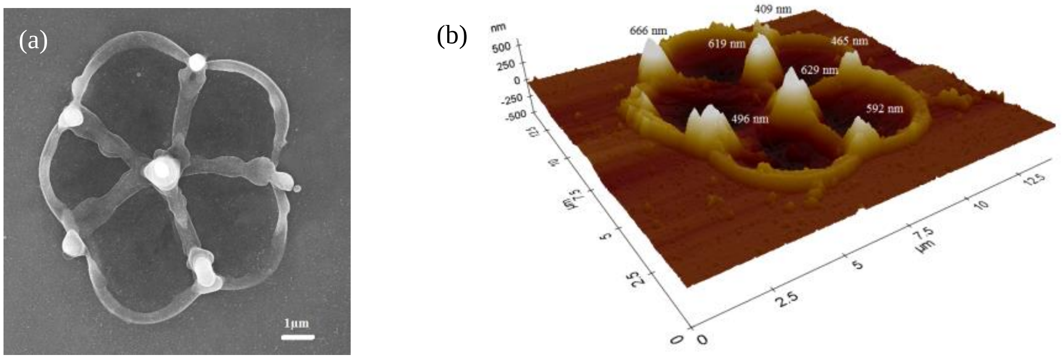

Figure S6. (a) The SEM image shows a 5-petal flower-shaped structure produced by a tuned q = -1 and detuned q = ½ plates. The structure consists of a single nanocone at the centre of the ablated region surrounded by 5 nanocones located at the same radial distance from the centre. (b) The AFM image of the 5-petal flower-shaped structure, shown in the Fig. 4b of the main text, with the central cone splitting into two due to detuning of the q = -1 plate. The heights of the nanocones vary from 400 nm to 670 nm.

References

[1] E. Karimi, B. Piccirillo, L. Marrucci, and E. Santamato, “Hypergeometric-Gaussian modes”, Opt. Lett. 34, 1225 (2009).

[2] L.Marrucci, E. Karimi, S. Slussarenko, B. Piccirillo, E. Santamato, E. Nagali, and F. Sciarrino, “Spin-to-orbital conversion of the angular momentum of light and itsclassical and quantum applications”, J. Opt. 13, 064001(2011).

[3] B. Piccirillo, V. D’Ambrosio, S. Slussarenko, L. Marrucci, and E. Santamato , “Photon spin-to-orbital angular momentum conversion via an electrically tunable q-plate”, APPLIED PHYSICS LETTERS 97, 241104 (2010).

[4] S. Baumann, D. Kalb, L. MacMillan, E. Galvez, “Propagation dynamics of optical vortices due to Gouy phase” Opt. Express, 17, 9818–9827 (2009).
